# Cloning and Functional Verification of Salt Tolerance Gene *HbNHX2* in *Hordeum brevisubulatum*

**DOI:** 10.3390/plants14233658

**Published:** 2025-11-30

**Authors:** Mingzhi Zhang, Mei Yang, Wenjie Zhao, Hang Yin, Xinyi Zhang, Bing Li, Muzhapaer Tuluhong, Baiji Wang, Shanshui Zheng, Guowen Cui

**Affiliations:** College of Animal Science and Technology, Northeast Agricultural University, Harbin 150030, China

**Keywords:** *Hordeum brevisubulatum*, membrane-bound Na^+^/H^+^ antiporters, *HbNHX2* gene, physiological mechanism, salt stress

## Abstract

A high salt environment seriously affects the physiological metabolism and yield of plants. *Hordeum brevisubulatum* (Trin.) Link has high biomass and important ecological, feeding and economic values, but its growth conditions have serious saline-alkali effects. The *NHX* gene family plays a vital role in regulating intracellular Na^+^/K^+^ balance, pH homeostasis, and vesicle and protein transport in plants. In this study, the *HbNHX2* gene was cloned from *Hordeum brevisubulatum* and functionally characterized through phenotypic, physiological, and molecular analyses in transgenic tobacco. Expression profiling revealed that *HbNHX2* was most abundant in spikes and least abundant in root tips, and the expression level was significantly induced under salt stress. Overexpression of *HbNHX2* led to decreased malondialdehyde (MDA) and superoxide anion (O^2−^) levels, while it enhanced the activities of superoxide dismutase (SOD), peroxidase (POD), and catalase (CAT). Additionally, the levels of glutathione (GSH), soluble proteins, proline, and chlorophyll were also increased. Several stress-responsive genes, including *CBL1*, *ERF2*, *BI-1*, *Cu/Zn-SOD*, *Mn-SOD*, *POD*, *GR1*, *KC1*, *TPK1*, *TIP*, *P5CS*, *BAS1*, *STN7* and *LTP1*, were significantly upregulated, while *SERK3B* was downregulated. These findings suggest that *HbNHX2* enhances plant salt tolerance by maintaining osmotic balance, scavenging reactive oxygen species (ROS), and regulating stress-responsive gene expression. This study provides new insights into the molecular mechanism of salt tolerance in *Hordeum brevisubulatum* and lays a foundation for breeding salt-tolerant forage crops.

## 1. Introduction

Globally, more than 900 million hectares of land—approximately 7% of the total terrestrial area—are affected by salinity and alkalinity [1]. The Songnen Plain, a major grassland region in Northeast China, represents a typical example of soda saline-alkaline soils [2]. These soils are characterized by high concentrations of alkaline salts such as sodium carbonate (Na_2_CO_3_) and sodium bicarbonate (NaHCO_3_), along with the accumulation of neutral salts, primarily sodium chloride (NaCl). Excessive salt accumulation imposes severe saline-alkali stress, significantly restricting plant growth and development in this region [3,4]. Short-term salt stress causes a rapid decline in plant water content, inhibiting growth and physiological functions [5]. Understanding plant adaptive mechanisms to saline environments is therefore essential for developing sustainable strategies to improve crop productivity.

Among the various salt-responsive pathways, NHX (Na^+^/H^+^ exchanger) genes have been widely recognized as key regulators associated with stress adaptation and plant development [6,7]. The NHX gene family is evolutionarily conserved across diverse plant species and exhibits characteristic transmembrane structural domains that determine its functional specificity [8]. NHX antiporters are crucial regulators of cellular osmotic adjustment by maintaining vacuolar turgor pressure, promoting water uptake, stabilizing intracellular pH, and ultimately regulating plant drought and salinity tolerance [9,10,11]. To date, NHX genes have been identified in various plant species, including *Arabidopsis thaliana* [12], rice [13], maize [14], *Brassica napus* [15], and wheat [16]. Numerous salt tolerance-related genes, including NHX family members, have also been developed as genetic resources for stress-resilient crop improvement [17].

Functional investigations have demonstrated that NHX overexpression is an effective strategy for enhancing plant stress resistance. For example, transgenic rice overexpressing *OsNHX1* exhibited significantly improved salt tolerance [14]. A genome-wide analysis in rice revealed 16 NHX orthologs and confirmed their involvement in drought responses, with *OsNHX7* and *OsNHX16* showing significantly higher expression in transgenic leaves compared with wild-type plants [18]. Overexpression of *NnNHX1* in tobacco also conferred enhanced salt tolerance in a dose-dependent manner [19], and *AtNHX1*-overexpressing Arabidopsis plants maintained vigorous growth under 200 mmol L^−1^ NaCl conditions [20]. Additionally, *GhNHX1* plays a key role in salt stress response in cotton [21], while *IbNHX2* expression is positively associated with drought tolerance in transgenic sweet potato [22]. Collectively, these studies confirm that NHX proteins act as critical determinants of plant resilience to salinity and drought stress. NHX genes are implicated not only in salt resistance but also in broader physiological processes such as cell expansion, vacuolar development, protein trafficking, reproductive growth, and overall biomass accumulation [23,24,25]. Transgenic experiments in multiple species have demonstrated that enhancing NHX gene expression improves plant growth performance and stress adaptability, supporting their strong application potential for crop genetic improvement [26]. Therefore, comprehensive elucidation of NHX molecular characteristics and functions remains a vital objective in stress biology research [27,28,29].

*Hordeum brevisubulatum*, a perennial grass species in the Poaceae family, is characterized by dense tillering, strong regenerative capacity, high forage yield, and notable tolerance to cold, drought, and salinity [30,31]. Its ecological and economic importance makes it an ideal candidate for molecular studies aimed at improving salt tolerance [32]. However, studies on NHX gene family characteristics and functional verification in *Hordeum brevisubulatum* remain insufficient, leaving gaps in understanding the molecular basis of its remarkable salt resilience. Although NHX genes are widely cloned and functionally identified in a variety of species, current research mainly focuses on cultivated plants or model species. However, little attention has been given to NHX genes from naturally salt-resilient germplasm, especially *Hordeum brevisubulatum*, which has evolved unique adaptive mechanisms under long-term saline–alkali environments. Therefore, exploring NHX gene characteristics and functions in *Hordeum brevisubulatum* may uncover novel allelic variations and distinct regulatory features that are absent in domesticated crops.

In this study, we performed systematic bioinformatics characterization, gene cloning, transgenic expression analysis, and physiological evaluation to investigate the functional role of *HbNHX2* in salt stress responses. The results will contribute to understanding NHX-mediated stress adaptation strategies in *Hordeum brevisubulatum* and provide important genetic resources for the development of salt-tolerant crops and efficient utilization of saline-alkali lands.

## 2. Results

### 2.1. Identification of HbNHX Family Members

**Figure 1:**
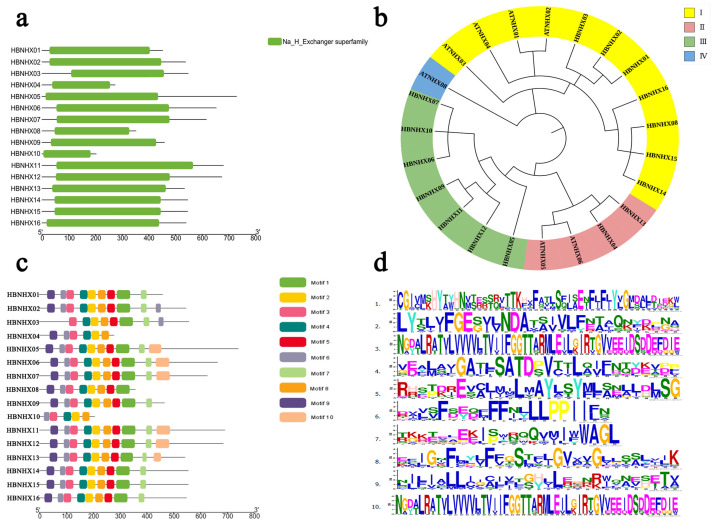
(**a**) Conserved domains of HbNHX family proteins; (**b**) phylogenetic tree of HbNHX gene family; (**c**) Conservative motif analysis of HbNHX family members; (**d**) logos of 10 motifs in HbNHX proteins.

Using transcriptome data from *Hordeum brevisubulatum*, a total of 16 NHX family members containing the complete NHX superfamily domain were identified and designated *HbNHX01* to *HbNHX16* (Figure 1a). Motif analysis showed that there were several highly conserved motifs in all members. In particular, motif-2, 3, 4, 8 showed consistency in all family members, which may be related to protein interaction or DNA binding The logo involved is shown in Figure 1d (Figure 1c,d).

The predicted proteins ranged from 203 to 729 amino acids, with molecular weights of 22.31–78.81 kDa and isoelectric points (pI) ranging from 4.88 to 9.61. Five members were predicted to be unstable, and all were hydrophobic proteins. Subcellular localization analysis predicted that these proteins were mainly localized to the inner membrane (Table S2).

Phylogenetic analysis was conducted by comparing the amino acid sequences of 16 *HbNHX* proteins with 7 Arabidopsis NHX proteins. The resulting phylogenetic tree (Figure 1b) classified the proteins into four groups. Group I included four Arabidopsis NHXs and seven *HbNHX* proteins (HbNHX01, 02, 03, 08, 14, 15, and 16); Group II contained one Arabidopsis NHX; Group III included seven HbNHX proteins (HbNHX05-HbNHX12); and Group IV consisted of two Arabidopsis and two HbNHX proteins (HbNHX04, 13).

### 2.2. Cloning and Characteristics of HbNHX2

**Figure 2:**
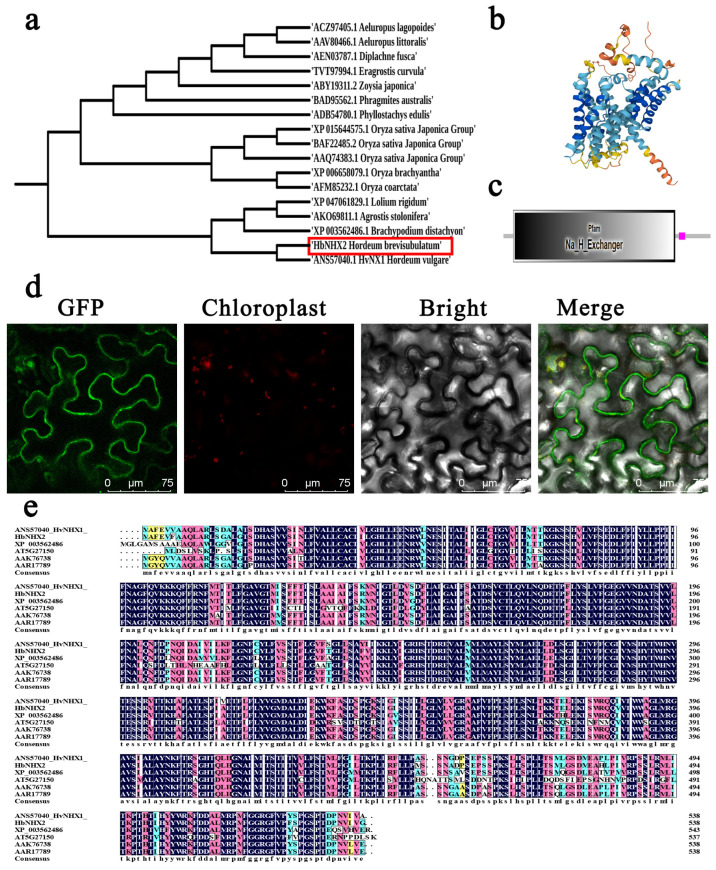
Amino acid sequence analysis and Subcellular localization of *HbNHX2*. (**a**) Phylogenetic tree of *HbNHX2*. (**b**) 3D protein model of *HbNHX2*. (**c**) Domains of *HbNHX2* protein. (**d**) Subcellular localization of *HbNHX2*. (**e**) Amino acid sequence alignment.

The full-length coding sequence (CDS) of *HbNHX2* was 1617 bp, encoding a 538-amino acid protein (GenBank accession: OQ596533). The predicted molecular weight was 59.28 kDa with a theoretical pI of 8.14. The average hydrophilicity index was 0.608, and the protein was classified as stable and hydrophobic. According to the phylogenetic tree, HbNHX2 is in the same branch as HvNHX1 in barley, indicating that these genes have the greatest similarity (Figure 2a). The predicted 3D structure from SWISS-MODEL was consistent with its secondary structure (Figure 2b). Multiple sequence alignment showed 96.67% identity between HbNHX2 and HvNHX1 from barley (Figure 2e). Secondary structure prediction revealed that *HbNHX2* contained approximately 45% α-helices, 5% β-turns, and 30% random coils. Subcellular localization prediction indicated that *HbNHX2* was most likely localized to the plasma membrane. Transient expression of *HbNHX2-GFP* in tobacco leaf epidermal cells confirmed its localization in both the plasma membrane and chloroplasts (Figure 2d).

### 2.3. Expression Pattern Analysis of HbNHX2

**Figure 3:**
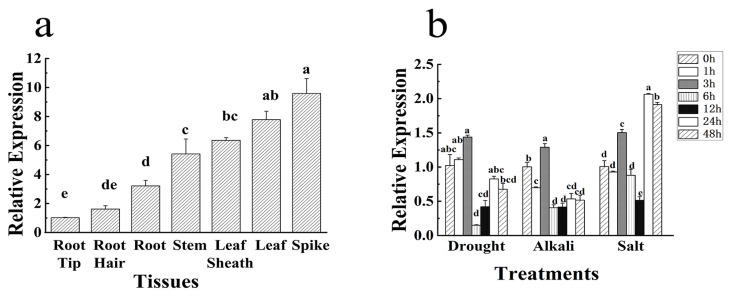
Expression pattern of *HbNHX2*. (**a**) The relative expression of *HbNHX2* gene in different tissues of *Hordeum brevisubulatum*. (**b**) The expression level of *HbNHX2* gene in leaf of *Hordeum brevisubulatum* under different stresses. The error bars represent the means ± student error values, and the different small letters on the error bars indicate significant differences among treatments at *p* < 0.05; the same applies below.

Quantitative expression analysis revealed that *HbNHX2* was highly expressed in the spike and least expressed in the root tip. Expression in the spike and stem was 9.39 times and 5.31 times higher than in the root hair, respectively (Figure 3a). In the leaves of *Hordeum brevisubulatum*, under drought stress, the expression of *HbNHX2* gene showed a trend of increasing first, then decreasing and then fluctuating, which was significantly up-regulated at 3 h of stress, reaching a peak (1.41 times). At 6 h, it began to decrease to the lowest level. At 48 h, *HbNHX2* gene was significantly up-regulated compared with that at 6 h, but it was still lower than that at 0 h. Under alkali stress, the expression level of *HbNHX2* gene showed a trend of first increasing, decreasing, then increasing, then decreasing and then stabilizing. The expression level of *HbNHX2* gene was the highest at 3 h, which was 1.28 times of that at 0 h, and reached the lowest level at 6 h (0.40 times). Under NaCl treatment, *HbNHX2* expression level in leaves showed a dynamic response. It initially increased at 3 h (1.49 times), decreased at 6 h, reached its lowest level at 12 h (0.51 times), and peaked at 24 h (2.05 times) (Figure 3b).

### 2.4. Genetic Transformation, Expression, and Phenotypic Analysis of Transgenic Tobacco

**Figure 4:**
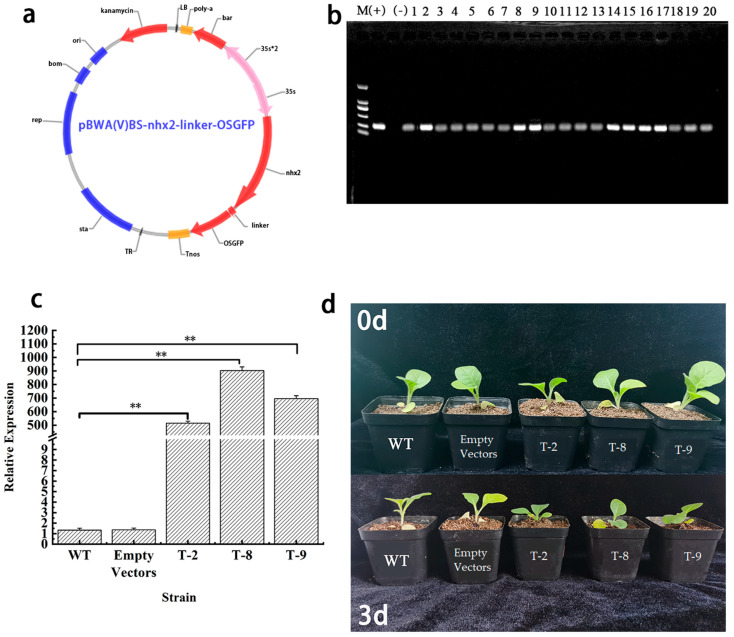
(**a**) pBWA(V) BS-HbNHX2-linker-OSGFP recombinant vector diagram. (**b**) Screening of *HbNHX2* gene gel map. (**c**) Expression level of *HbNHX2* in transgenic tobacco lines. (**d**) Phenotypic changes of 0 d and 3 d under 200 mM NaCl treatment. WT: wild type. T-2, T-8, and T-9: transgenic tobacco lines 2, 8, and 9, respectively. The same applies below. **: *p* < 0.01.

To investigate the role of *HbNHX2* in salt tolerance, an overexpression vector (pBWA(V)BS-nhx2-linker-OSGFP) was constructed and transformed into tobacco (*Nicotiana tabacum* L. cv. K326) Among the transformed lines, T-2, T-8 and T-9 were selected based on PCR and qRT-PCR validation. These lines showed significantly higher *HbNHX2* expression compared to WT plants (*p* < 0.01). Under 200 mM NaCl treatment, the non-transgenic tobacco lines showed an obvious yellowing phenomenon, indicating that the transgenic tobacco had resistance to salt stress (Figure 4). The empty-vector line showed no significant differences from WT under either or salt treatment conditions, confirming that the phenotypic changes observed in the overexpression lines were specifically due to *HbNHX2* expression.

### 2.5. Physiological Response of Transgenic Tobacco Under Salt Stress

**Figure 5:**
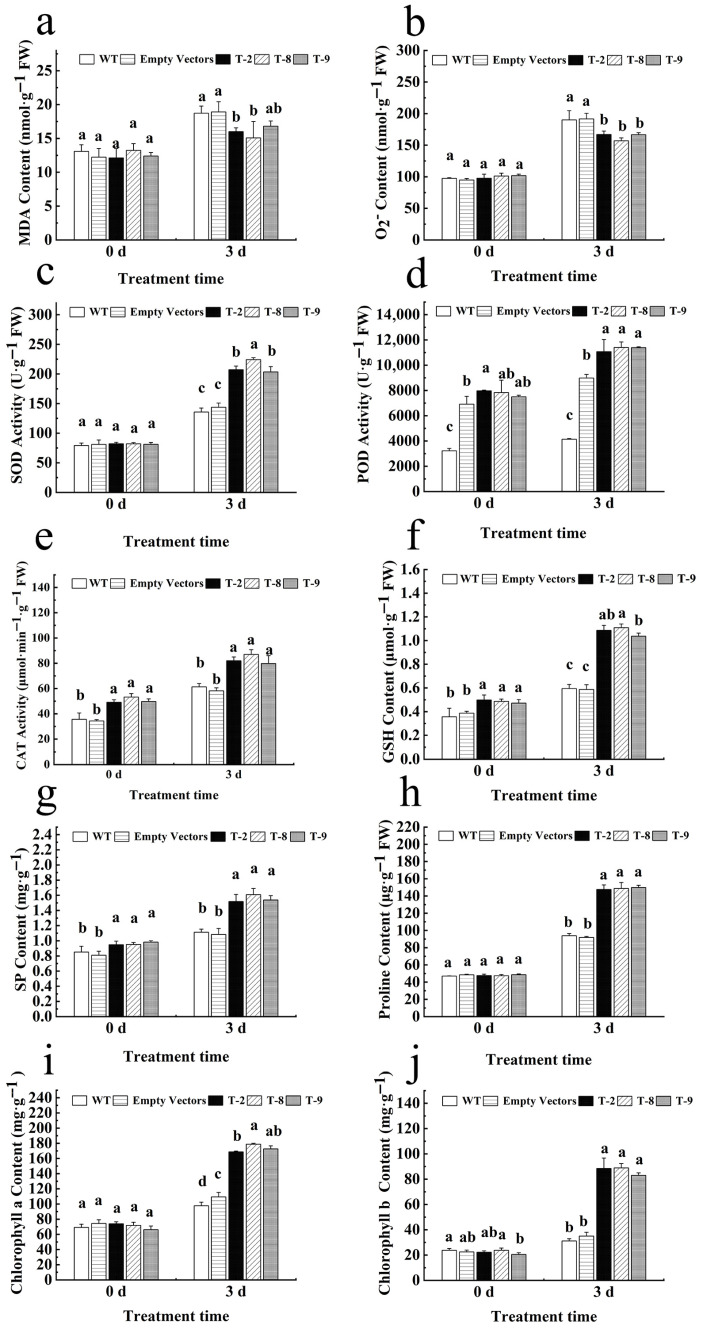
The changes inphysiological indexes in transgenic tobacco under salt stress. (**a**) MDA content; (**b**) O_2_^−^ content; (**c**) SOD activity; (**d**) POD activity; (**e**) CAT activity; (**f**) GSH content; (**g**) soluble protein content; (**h**) proline content; (**i**) chlorophyll *a* content; (**j**) chlorophyll *b* content. Data are presented as mean ± SD (*n* = 3 biological replicates). Different letters indicate significant differences among WT, EV, and OE lines (*p* < 0.01, one-way ANOVA followed by Tukey’s test).

Physiological assays revealed that under 200 mM NaCl stress, transgenic lines overexpressing *HbNHX2* showed significantly lower levels of MDA and O_2_^−^, and higher level of SOD compared to WT and empty vector controls. Activities of antioxidant enzymes (POD, CAT) were significantly enhanced, although POD was significantly higher in the empty strain than in WT, POD activity in the OE strain was significantly higher than that in the empty strain. The overexpression lines also displayed increased levels of GSH, soluble protein, proline, and chlorophyll a and b, both under salt-stress conditions (Figure 5), These results suggest the overexpression of *HbNHX2* increased the plant level of improved antioxidant capacity, osmotic adjustment, and photosynthetic efficiency under salt stress conditions.

### 2.6. Expression of Stress-Responsive Genes in Transgenic Tobacco

**Figure 6:**
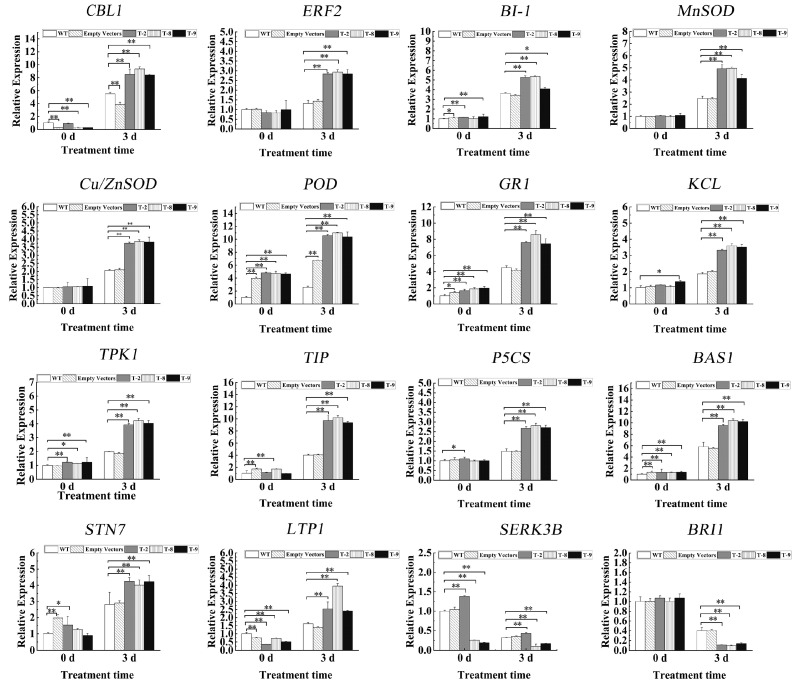
Expression patterns of stress-responsive genes in tobacco overexpressing *HbNHX2* under salt stress. *CBL1*: *Calcineurin B-like 1*. *ERF2*: Ethylene-Responsive Factor. *BI-1*: Bax inhibitor-1. *MnSOD*: superoxide dismutase [Mn]. *Cu/Zn-SOD*: superoxide dismutase [Cu-Zn] *POD*: peroxidase. *GR1*: protein gamma response 1. *KC1*: potassium channel KAT3-like 1. *TPK1*: Two-pore potassium channel 1-like 1. *TIP*: aquaporin TIP-type. *P5CS*: delta-1-pyrroline-5-carboxylate synthase. *BAS1*: 2-Cys peroxiredoxin. *STN7*: Serine/Threonine kinase domain protein. *LTP1*: lipid-transfer protein 1. *SERK3B*: Somatic Embryogenesis Receptor-like Kinase 3B. *BRI1*: brassinosteroid LRR receptor kinase. * indicates that the *p* value is less than 0.05, and ** indicates that the *p* value is less than 0.01.

To further explore the molecular mechanisms of *HbNHX2*-mediated salt tolerance, the expression of 16 stress-related genes was evaluated in transgenic and WT plants under 200 mM NaCl treatment (Figure 6). Under normal conditions, there was no significant difference in the expression levels of *ERF2*, *MnSOD*, *Cu/Zn-SOD* and *BRI1* in each strain. After stress treatment, the expression levels in OE strains were significantly different from those in WT and empty strains. *ERF2*, *MnSOD*, and *Cu/Zn-SOD* were significantly up-regulated, and *BRI1* was significantly down-regulated. The expression levels of *KC1*, *CBL1*, and *P5CS* were inconsistent in each line under normal conditions, but they were significantly up-regulated in OE lines after stress. Although the expression levels of *BI-1*, *POD*, *GR1*, *TIP*, *BAS1*, *STN7* and *LTP1* in the empty lines were significantly different from those in WT under normal conditions, their expression levels in OE lines were significantly higher than those in WT and empty lines after stress treatment. These results indicate that overexpression of *HbNHX2* can regulate stress-related genes to respond to salt stress without being affected by the vector.

## 3. Discussion

### 3.1. HbNHX2 and the NHX Gene Family in Plant Salt Tolerance

Na^+^/H^+^ antiporters (NHXs) are key membrane-bound transporters widely distributed in eukaryotes that maintain ion and pH homeostasis, thereby enabling plants to cope with diverse abiotic stresses [33]. They exchange cytosolic Na^+^ for H^+^ across cellular membranes and participate in multiple biological processes, including vacuolar ion compartmentalization, vesicle trafficking, cell expansion, and osmotic regulation [34]. In this study, *HbNHX2* was cloned from *Hordeum brevisubulatum*, a halophytic species known for its remarkable salt tolerance. The protein shared high sequence similarity with *HvNHX2* from barley, suggesting evolutionary conservation and functional relevance.

Expression profiling showed that *HbNHX2* was highly expressed in stems and roots and was significantly induced under both salt and low-temperature stresses, implying a role in stress perception and ion homeostasis regulation, These results are consistent with previous reports on *AtNHX1* in *Arabidopsis* and *OsNHX1* in rice, both of which are strongly upregulated under salinity and contribute to enhanced Na^+^ sequestration and K^+^ retention [33,35,36]. Therefore, *HbNHX2* may function as an important vacuolar or endosomal Na^+^/H^+^ antiporter in the halophytic adaptation of *Hordeum brevisubulatum*.

### 3.2. Functional and Physiological Characterization of HbNHX2 Was Studied Using Genetic Transformation

Subcellular localization analysis demonstrated that *HbNHX2* was mainly distributed on the plasma membrane and chloroplast envelope, which is consistent with class II NHX-type proteins localized to endomembrane systems [37]. Dual localization of *HbNHX2* to both the plasma membrane and chloroplast is particularly intriguing and may imply its multifaceted function in ion homeostasis under salt stress. NHX family members localized on the plasma membrane have been reported to participate in Na^+^ extrusion and K^+^ uptake to maintain cytosolic ion balance [23]. In contrast, chloroplast-associated NHX proteins are known to regulate stromal K^+^ retention, thylakoid lumen pH, and photosynthetic efficiency [38,39]. The dual localization of *HbNHX2* therefore suggests that it may coordinate ion distribution across multiple cellular compartments. Under salt stress, a plasma membrane-localized pool of *HbNHX2* could prevent cytosolic Na^+^ toxicity by excluding excess Na^+^, while the chloroplast-localized fraction might help sustain adequate K^+^ levels for optimal enzyme activity and photochemical stability, thereby mitigating chloroplast dysfunction and leaf chlorosis. These functions align well with the enhanced growth performance and alleviated photosynthetic inhibition observed in the transgenic plants. Future work combining ion-specific probes or subcellular fractionation will help clarify whether *HbNHX2* modulates Na^+^ and K^+^ partitioning between the cytosol and chloroplast to confer salt tolerance more precisely. To further elucidate its function, *HbNHX2* was overexpressed in K326. The transgenic plants displayed significantly enhanced salt tolerance compared with wild-type and empty-vector controls (Figure 4d).

Under NaCl stress, *HbNHX2*-overexpressing lines maintained significantly lower malondialdehyde (MDA) and superoxide (O_2_^−^) levels, indicating reduced lipid peroxidation and oxidative membrane damage [40].This protection was associated with elevated activities of major antioxidant enzymes, including superoxide dismutase (SOD), peroxidase (POD), and catalase (CAT), and increased glutathione (GSH) content. Such enhancement of the antioxidant system suggests that *HbNHX2* indirectly contributes to ROS detoxification, reducing oxidative stress in plant cells [41]. The empty-vector lines behaved similarly to WT in growth and physiological responses, indicating that the phenotype enhancement resulted from *HbNHX2* expression rather than vector effects, consistent with previous reports on NHX overexpression.

Moreover, transgenic plants accumulated higher levels of soluble protein and proline, which serve as osmoprotectants and help maintain osmotic potential under salt stress [42], Elevated chlorophyll a and b contents further indicated that photosynthetic efficiency and pigment stability were better maintained in transgenic lines than in controls. Together, these findings demonstrate that *HbNHX2* not only contributes to ion and osmotic balance but also enhances redox homeostasis and photosynthetic stability, resulting in improved physiological resilience under salinity.

NHX-type Na^+^/H^+^ antiporters primarily contribute to ion homeostasis by reducing cytosolic Na^+^ and maintaining cellular K^+^ levels under salt stress, which are essential for ROS detoxification and enzyme stability [43]. Improved ion balance can indirectly attenuate ROS generation in chloroplasts and mitochondria, thereby reducing oxidative damage. Meanwhile, increasing evidence suggests that NHX activity may also modulate ROS signaling pathways, influencing the transcription of antioxidant-related genes through Ca^2+^ or MAPK signaling crosstalk [39,41]. Thus, the enhanced antioxidant capacity observed in *HbNHX2* plants is likely the result of coordinated effects of improved ion homeostasis and stress-responsive ROS signaling. To further strengthen this mechanistic model, future work could investigate the expression of ROS-related regulatory genes and quantify ROS levels together with ion contents (e.g., Na^+^/K^+^ ratios) to establish correlations among ion transport, ROS homeostasis, and transcriptional responses [44]. Such evidence would help clarify whether *HbNHX2* confers salt tolerance primarily via direct ROS signaling modulation, indirect ionic regulation, or both.

In various plants, the compartmentalization of Na^+^ and the maintenance of K^+^ homeostasis are crucial for salt stress tolerance. For instance, overexpression of the *McNHX1* gene in tobacco significantly enhances vacuolar Na^+^ compartmentalization, thereby improving salt tolerance in sensitive plants [45]. Similarly, overexpression of *OsNHX1* in *Oryza sativa* L. strengthens Na^+^ compartmentalization in the roots, reducing Na^+^ transport to the shoot while maintaining K^+^ levels in the leaves [13]. In tobacco, overexpression of *TaNHX3* in *Triticum aestivum* L. is not only promoted Na efflux but also helps retain K induced, contributing to ion homeostasis [46]. In *Ricinus communis* L., compared with the control group, the plants overexpressing *SbNHX1* showed lower Na^+^ content and higher K^+^ ion accumulation, and generally showed higher K^+^/Na^+^ [47] (Table S3).

Comparative analysis suggests that *HbNHX2* functions similarly to NHX genes from halophytes and salt-tolerant algae, mainly by maintaining Na^+^/K^+^ homeostasis and mitigating oxidative stress under salinity [48]. This indicates a conserved protective mechanism across salt-adapted species. Meanwhile, the observed effects on antioxidant regulation and signaling dynamics may reflect species-specific optimization for the extreme saline environment of *Hordeum brevisubulatum*.

Potential trade-offs should also be considered. Constitutive NHX activation may affect cellular pH gradients, energy cost, or hormone balance, potentially limiting growth when stress is absent [33]. Therefore, conditional expression strategies or long-term performance evaluation would be valuable for future applications.

### 3.3. Transcriptional Regulation of Stress-Responsive Genes in HbNHX2-Overexpressing Plants

Quantitative RT-PCR analysis revealed that *HbNHX2* overexpression triggered broad transcriptional reprogramming in tobacco, affecting multiple stress-responsive pathways. Upregulation of *CBL1*, a calcium sensor involved in abiotic stress signaling, indicates that *HbNHX2* may interact with calcium-dependent signaling cascades to facilitate salt tolerance [49,50,51,52]. Similarly, *ERF2*, an ethylene-responsive transcription factor, and *BI-1*, an anti-apoptotic gene suppressing ROS-induced cell death, were significantly upregulated, enhancing cellular adaptation to oxidative and ionic stresses [53]. Genes related to ion transport (*KC1*, *TPK1*) and osmotic regulation (*TIP*, *P5CS*) were also strongly induced. These genes are essential for maintaining K^+^ absorption, vacuolar ion storage, and proline biosynthesis, collectively supporting osmotic adjustment and ion balance [54,55,56]. The enhanced expression of *Cu/Zn-SOD*, *MnSOD*, and *NtPOD* was consistent with higher enzyme activities observed physiologically, reflecting stronger ROS-scavenging capacity. In addition, the upregulation of *BAS1* and *STN7* suggests that *HbNHX2* may stabilize chlorophyll content and maintain photosynthetic electron transport under salinity [57,58,59,60,61].

Conversely, *SERK3B* and *BRI1*, both known to negatively regulate stress resistance, were significantly downregulated in *HbNHX2*-overexpressing plants. The downregulation of *SERK3B* and *BRI1* in the transgenic lines is intriguing and may indicate an adaptive adjustment of brassinosteroid (BR) signaling under salt stress. BRI1 and its co-receptor SERK3/BAK1 are essential for BR perception and growth promotion, but several studies have shown that partial suppression of BR signaling can enhance abiotic stress tolerance by redirecting resources from cell expansion toward defense and stress protection. Reduced BR signaling may also enhance ABA sensitivity, regulate the expression of ion transporters, and modulate ROS-scavenging enzymes. Under saline conditions, lower BRI1–SERK3B activity could help maintain ion and osmotic homeostasis by reducing Na^+^ influx through decreased transpiration, while promoting protective pathways that stabilize membranes and chloroplast function. Thus, the observed downregulation of these genes may reflect a strategic reprogramming that balances growth inhibition with improved stress resilience. Further experiments, such as exogenous BR application or BR-responsive gene analysis, would clarify whether suppression of BR signaling directly contributes to the enhanced salt tolerance observed here [62,63].

**Figure 7:**
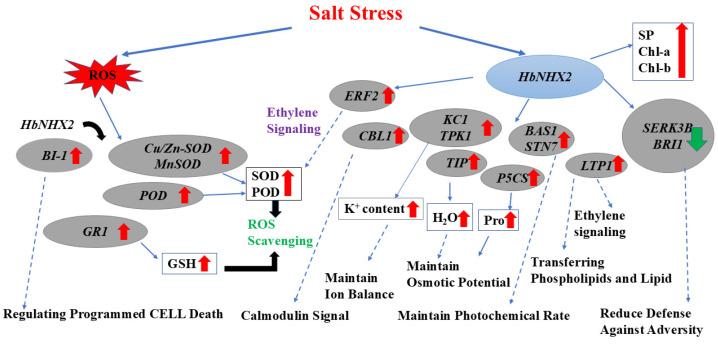
Molecular mechanism of *HbNHX2* in response to salt stress. The red arrow indicates the up-regulation of genes or the increase in physiological indicators, and the green arrow indicates the down-regulation of genes. Possible regulatory mechanisms are represented by dotted lines.

These integrated physiological and molecular responses collectively form the basis of a proposed model for *HbNHX2*-mediated salt tolerance (Figure 7). It activates osmotic adjustment through increased accumulation of soluble proteins and proline. Enhanced antioxidant enzyme activities (SOD, POD, CAT) and elevated GSH levels contribute to effective ROS detoxification, reducing oxidative membrane damage. At the molecular level, *HbNHX2* overexpression induces transcription of *CBL1*, *ERF2*, *BI-1*, *NtKC1*, *NtTPK1*, *TIP*, *P5CS*, and antioxidant-related genes, while repressing *SERK3B* and *BRI1*. This coordinated regulation enhances Ca^2+^ signaling, ethylene response, osmotic regulation, and redox balance, forming a multilayered defense network. The synergistic regulation of these pathways enables plants to maintain physiological and metabolic stability in high-salinity environments.

In conclusion, *HbNHX2* confers salt tolerance by integrating ionic, osmotic, and oxidative stress responses at both physiological and transcriptional levels. These findings expand our understanding of NHX-mediated stress adaptation and provide a valuable genetic resource for improving crop resilience through molecular breeding and transgenic approaches.

## 4. Materials and Methods

### 4.1. Plant Growth and Treatment

In this study, *Hordeum brevisubulatum* (Trin.) Link cv. Saertu was used as the plant material. Mature and uniform seeds of *Hordeum brevisubulatum* were surface-sterilized through immersion in 10% NaClO for 1 min, followed by three rinses with distilled water, treatment with 70% ethanol for 2 min, and finally washed 4–5 times with sterile water. The sterilized seeds were cultured on germination medium in an incubator. After 10 days, seedlings were transplanted to plastic pots filled with vermiculite and maintained in a growth chamber at 25 ± 2 °C and 65% ± 2% relative humidity, under a 16 h light/8 h dark photoperiod. Plants were irrigated with 1/10 Hoagland nutrient solution. Most of the 30-day-old *Hordeum brevisubulatum* seedlings were subjected to 15% PEG-6000, 150 mM NaCl, and 150 mM NaHCO_3_ for 0 (control), 3, 6, 12, 24, and 48 h. Leaf samples were frozen in liquid nitrogen and stored at −80 °C for subsequent analysis. Each treatment included three biological replicates. The remaining plants were further cultivated until they began to spike. The spikes, leaves, leaf sheaths, stems, roots, root hairs, and root tips of *Hordeum brevisubulatum* were collected and frozen in liquid nitrogen for storage at −80 °C for tissue-specific expression analysis.

### 4.2. Identification of HbNHX Gene Family Members

NHX amino acid sequences of *Arabidopsis thaliana* were retrieved from the TAIR database (http://arabidopsis.org/) (accessed on 1 October 2025). Using the transcriptome data of *Hordeum brevisubulatum* from previous studies, a local BLAST database (ncbi-blast-2.17.0 was constructed to identify homologs according to Arabidopsis NHX sequences. Candidate NHX proteins were verified using Batch-CDD (https://www.ncbi.nlm.nih.gov/cdd) (accessed on 1 October 2025). A total of 16 putative *HbNHX* genes were identified, after excluding sequences lacking the NHX domain.

Physicochemical properties and subcellular localization of HbNHX family members were predicted using ExPASy (https://web.expasy.org/protparam/) (accessed on 1 October 2025) and CELLO (http://cello.life.nctu.edu.tw/) (accessed on 1 October 2025), respectively. Conserved motifs were analyzed using MEME Suite (http://meme-suite.org/) (accessed on 1 October 2025) and visualized with TBtools v2.096. Homology analysis was conducted via blast using the NCBI database, and a phylogenetic tree was constructed using MEGA11.0 software with the neighbor-joining method.

### 4.3. Isolation and Cloning of HbNHX2

Total RNA was extracted from healthy *Hordeum brevisubulatum* seedlings using an Ultrapure RNA Kit (CWBIO, Beijing, China), and RNA integrity and concentration were assessed with a NanoPhotometer (Implen, Munich, Germany). First-strand cDNA synthesis was performed using the HiScript II Q Select RT SuperMix for qPCR (+gDNA wiper) (Vazyme, Nanjing, China). The CDS of *HbNHX2* was amplified using primers (Table S1) designed with Primer Express 5.0 based on the *Hordeum vulgare HvNHX1* gene sequence (ANS57040.1). The PCR products of *HbNHX2* were was inserted into the pCE2 TA/Blunt-Zero vector (Vazyme, Nanjing, China) using the ClonExpress R II One Step Cloning Kit (Vazyme, Nanjing, China) and sequenced by RuiBiotech Co. (Harbin, China). The conserved domain and protein structure of *HbNHX2* were predicted using NCBI-CDD, SOPMA (http://pbil.ibcp.fr) (accessed on 1 October 2025), and SWISS-MODEL (https://swissmodel.expasy.org/) (accessed on 1 October 2025) [64,65,66].

### 4.4. Expression Pattern Analysis of Tissue Specificity and Stress Response of HbNHX2 Gene 

Real-time quantitative PCR (qRT-PCR) was used to analyze tissue-specific and stress-responsive expression patterns of *HbNHX2*. Total RNA from each sample was reverse-transcribed using the HiScript II Q Select RT SuperMix for qPCR (+gDNA wiper); (Vazyme, Nanjing, China). qRT-PCR was performed using ChamQ Universal SYBR qPCR Master Mix (Vazyme, Nanjing, China) on a Quantagene q225 Real-Time PCR system (Novogene, Nanjing, China). Primer sequences are listed in Table S1. The *HvActin* was used as the internal reference gene, and relative expression levels were calculated using the comparative cycle threshold (Ct) method (2^−ΔΔCt^) [67].

### 4.5. Plant Transformation and Generation of Transgenic Tobacco

The coding sequence of *HbNHX2* was cloned into the pBWA(V)BS-ccdB-OSGFP vector using BsaI/Eco31I restriction enzymes, generating the pBWA(V)BS-HbNHX2-linker-OSGFP overexpression vector. The recombinant plasmid was introduced into *Agrobacterium tumefaciens* strain *EHA105* to perform genetic transformation for wild-type (WT) of tobacco. Transgenic tobacco was selected on glyphosate-containing medium (15 mg/L), and positive transformants were identified using PCR with *Bar* gene specific primers (Table S1). Homozygous lines were confirmed with qRT-PCR using qPCR-HbNHX2 primers, with *NtActin* as the reference gene (Table S1). The homozygous linesT-2, T-8, and T-9 were selected for subsequent experiments.

### 4.6. Subcellular Localization of HbNHX2 Protein

Tobacco (*Nicotiana benthamiana)* seeds were germinated in vermiculite and irrigated with 1/10 Hoagland solution. Plants at the 4–5 true leaf stage were used for transient expression experiments.

The coding sequence of *HbNHX2* without a termination codon was cloned into the pBWA(V)BS-ccdB-OSGFP vector using BsaI/Eco31I restriction enzymes to generate the pBWA(V)BS-HbNHX2-linker-OSGFP fusion construct under the control of the 35S promoter. The recombinant plasmid and the control vector (35S::OSGFP) were transformed into Agrobacterium tumefaciens strain GV3101 and transiently expressed in tobacco leaves through agroinfiltration. After incubation for 48–72 h, GFP fluorescence was observed using a confocal laser scanning microscope (Leica TCS SP2 AOBS, Shanghai, China). The localization of the HbNHX2-GFP fusion protein was analyzed by comparing the GFP signal.

### 4.7. Physiological Index of Homozygous Lines Subjected to Salt Stress

A total of 400 seeds from transgenic and WT tobacco lines were germinated on 1/2 MS medium after vernalization at 4 °C for 2 days. After germination, seedlings were transplanted into vermiculite-filled pots and grown under controlled conditions (25 °C). After 15 days, uniform and healthy seedlings were subjected to 200 mM NaCl stress for 3 days. Leaf samples before and after treatment were harvested, frozen in liquid nitrogen, and stored at −80 °C for further physiological and molecular analyses. Each treatment included three biological replicates, with each replicate containing three plants.

The contents of malondialdehyde (MDA) were determined using the thiobarbituric acid (TBA) method, and the generation rate of superoxide anion (O_2_^−^) was measured using the hydroxylamine method. Superoxide dismutase (SOD) activity was assayed using the nitroblue tetrazolium (NBT) photoreduction inhibition method, peroxidase (POD) activity with the guaiacol method, and catalase (CAT) activity by monitoring the decrease in absorbance at 240 nm (A240 method). Reduced glutathione (GSH) content was determined using the 5,5′-dithiobis-(2-nitrobenzoic acid) (DTNB) method, soluble protein content was measured with the Bradford assay, and proline content was determined using the acidic ninhydrin method [68,69,70,71]. Chlorophyll a, b, and total chlorophyll contents were quantified using the acetone extraction method. were measured using commercial assay kits (Suzhou Keming Biotechnology Co., Ltd., Suzhou, China) following the manufacturer’s instructions. Absorbance values were determined using a k6600-a (Beijing Kaiao Technology Development Co., Ltd., Beijing, China) tmna microplate reader. All physiological measurements were based on at least three independent biological replicates (each replicate containing five plants per line).

### 4.8. Expression Analysis of Stress-Responsive Gene Subjected to Salt Stress

To further elucidate the molecular mechanisms underlying *HbNHX2*-mediated salt tolerance, we selected a set of marker genes representing major stress-responsive pathways. These included genes involved in ion homeostasis and signal transduction (*CBL1*, *KC1*, *PK1*, *SERK3B*, *BRI1*, *BAS1*), cell death and stress protection (*BI-1*), ROS scavenging (*MnSOD*, *Cu/Zn-SOD*, *POD*, *GR1*), osmotic regulation (*P5CS*), membrane water transport (*TIP*), lipid transfer and membrane stability (*LTP1*), photosynthetic protection (STN7), and ethylene-dependent stress signaling (*ERF2*). These genes have been widely reported to participate in salt response networks in plants. Their expression patterns were therefore used to evaluate whether *HbNHX2* overexpression enhances salt tolerance through coordinated regulation of ion homeostasis, ROS balance, and stress signaling cascades.

Seventeen stress-related genes were analyzed in WT and transgenic tobacco under salt stress. qRT-PCR was performed using *NtActin* as an internal reference. Primer sequences are listed in Table S1.

### 4.9. Data Analysis

The experiments were replicated three times using the same methodology and all the data are expressed as the means ± standard deviation. Gene expression and physiological data were statistically analyzed using Excel 2019 and SPSS 22.0. Graphs were generated and visualized using Origin 2018, TBtools v2.096 and PowerPoint 2019. Significant differences between treatments were determined using one-way ANOVA and independent-samples *t*-tests (α = 0.05 or 0.01, as indicated).

## 5. Conclusions

In this study, the 16 members of the *HbNHX* family in *Hordeum brevisubulatum* were identified, and the *HbNHX2* gene was cloned and functionally characterized. The gene encodes a stable and hydrophobic protein localized to the plasma membrane and chloroplast. Its expression is highest in spikes and is induced to salt and low-temperature stress. Transgenic tobacco plants overexpressing *HbNHX2* showed enhanced salt tolerance reflected by the improved antioxidant capacity and osmotic regulation. Moreover, *HbNHX2* influenced the expression of stress-related genes involved in ion transport, osmotic adjustment, and oxidative defense. Overall, *HbNHX2* plays a key role in plant salt stress adaptation and represents a promising candidate for improving salt tolerance in forage and crop species.

## Data Availability

The original contributions presented in this study are included in the article/Supplementary Material. Further inquiries can be directed to the corresponding authors.

## Supplementary Materials

The following supporting information can be downloaded at: https://www.mdpi.com/article/10.3390/plants14233658/s1, Table S1. List of primers in this study. Table S2. Physicochemical properties and subcellular localization of HbNHXs family protein. Table S3. The function of NHX gene in different plants and its effect on Na^+^ and K^+^ homeostasis under salt stress.

## Abbreviations

The following abbreviations are used in this manuscript:

MDA	Malondialdehyde
O_2_^−^	Superoxide anion
SOD	Superoxide dismutase
POD	Peroxidase
CAT	Catalase
ROS	Reactive oxygen species
GSH	Glutathione
K326	*Nicotiana tabacum* L. cv. K326
*CBL1*	*Calcineurin B-like 1*
*ERF2*	Ethylene-Responsive Factor
*BI-1*	Bax inhibitor-1
*MnSOD*	Superoxide dismutase [Mn]
*Cu/Zn-SOD*	Superoxide dismutase [41Cu-Zn]
*POD*	Nicotiana tabacum Peroxidase
*GR1*	Protein gamma response 1.
*KC1*	Potassium channel KAT3-like 1.
*TPK1*:	Two-pore potassium channel 1-like 1
*TIP*	Aquaporin TIP-type
*P5CS*	Delta-1-pyrroline-5-carboxylate synthase
*BAS1*	2-Cys peroxiredoxin
*STN7*	Serine/Threonine kinase domain protein
*LTP1*	Lipid-transfer protein 1.
*SERK3B*	Somatic Embryogenesis Receptor-like Kinase 3B
*BRI1*	Brassinosteroid LRR receptor kinase
