# Supplementary material for: Cloning and Functional Verification of Salt Tolerance Gene HbNHX2 in Hordeum brevisubulatum
Source: Plants (Basel). 2025 Nov 30;14(23):3658. doi: 10.3390/plants14233658 (PMC12694128; doi:10.3390/plants14233658)
Supplement: Supplementary file 1 [file plants-14-03658-s001.zip › plants-3985630-supplementary.pdf]

Supplementary Materials

Table S1. List of primers in this study.

Primer name	Forward primer (5' to 3')	Reverse primer (5' to 3')
HbNHX2	ATGGCGTTCGAAGTGATTG	TCATCCCACGATTACGTTTG
HbNHX2-TE	GAGAGAACACGGGGGACTTTGCAAC ATGGCGTTCGAAGTGATTGCGGC	CGGCCGCTGTACATCCCACGATTAC GTTTGGATCGGTG
HbNHX2-OE	CGTAATCGTGGGATGTACAGCGGCC GCTTCAGG	AGAGTTCCTCGCCCTTCACGATACAA ATTGCTGCGGCAGCCGATCCAG
BAR277	TGGGCAGCCCGATGACAGCGACCAC	ACCGAGCCGCAGGAACCGCAGGAGT
HvActin	CCACGAGACGACCTACAAC	CACTGAGCACGATGTTTCC
NtActin	GCTTTCTTCGTCCCATCA	CCCCAAGTACCCTCGTAT
qPCR-HbNHX2	GGATTGCTCAGTGCATTCGTC	AGTCTCGGCGATGAAGGACAAG
qPCR-CBL1	GCCTCACAAGAAGAGAAGATCA	AACTTCATCTCAGACTCGCATAG
qPCR-BI-1	CTTTGGAACATTGGTGGCTTAC	CCATCAGAAGTGCTATCCTCTTT
qPCR-KC1	TTTGCCATGAGGGATGCGAT	TGATCTTGTGTCCCGGCTTC
qPCR-Hxk3	TTCATCAACCTCCTGGTAAGC	CCTTTCGTCCACCTGATAAGAG
qPCR-SERK3B	CCTTCTTCAGGTGTGGGCAA	TCGATGCACAGCCATGCTAA
qPCR-BRI1	TGCTTGGAGAGCTTGACCTG	GAGACTTCTGGTGCTGGCTT
qPCR-ERF2	GCTACCTCCGACGAGTTTCA	ATCCGGCAGTTAAAGCGTCA
qPCR-TIP	TCAAGCACCACAGTCATCTCG	TTGTGTGCATGCTCTGGATCT
qPCR-BAS1	CTCTCTTCTCGCGTTGCTCA	TCACCTAGCCCACCAGACTT
qPCR-STN7	CCGGGCTACACAACAAGACT	AAGCCGAAGAGCTGATGCAA
qPCR-MnSOD	AGCAGACGGACCTTAGCAAC	GGCTCCAGTGCTCCATAGTC
qPCR-Cu/Zn-SOD	AGCAGCAGTGAAGGTGTTAG	GGCCAGAGACATTTCCAGTAA
qPCR-POD	AGGCAACGTACTGATGCTCG	ACGACGGACCTTTAGCCAAG
qPCR-P5CS	TGGAAGATTGGGAGCGCTTT	CCGCGTACTGATAGCGTCAT
qPCR-GR1	ACCTCCAACTTCAAGTGCCC	GATGCGACCTGGTATCCCTC
qPCR-Ltp1	GGCGTTAAGGCTCTGGTGAA	CTGGACCTTGGAGCAGTCAG

Table S2. Physicochemical properties and subcellular localization of HbNHXs family protein

Gene name	Peptide residue （aa）	MW (kD）	pI	instability index (II)	GRAVY	Aliphatic index	Subcellular localization
HBNHX01	452	49.81	6.79	29.65	0.757	117.99	Inner Membrane
HBNHX02	538	59.32	8.14	34.83	0.605	110.72	Inner Membrane
HBNHX03	548	60.53	9.61	48.93	0.158	91.81	Inner Membrane
HBNHX04	277	29.85	5.50	49.35	0.895	117.55	Inner Membrane
HBNHX05	729	78.81	5.89	41.09	0.06	85.34	Outer Membrane<br>Inner Membrane
HBNHX06	653	71.29	6.09	35.19	0.247	103.19	Inner Membrane
HBNHX07	616	67.97	9.31	35.41	0.253	100.37	Inner Membrane
HBNHX08	352	38.66	5.71	28.65	0.847	130.17	Inner Membrane
HBNHX09	459	50.50	8.43	33.57	0.791	123.66	Inner Membrane
HBNHX10	203	22.31	4.88	21.48	0.85	121.53	Inner Membrane
HBNHX11	680	74.26	6.13	40.98	0.205	103.71	Inner Membrane
HBNHX12	674	73.08	6.41	35.16	0.319	105.19	Inner Membrane
HBNHX13	534	58.41	4.97	45.89	0.458	101.91	Inner Membrane
HBNHX14	546	59.56	7.69	30.65	0.657	118.72	Inner Membrane
HBNHX15	546	59.57	7.69	30.79	0.667	119.43	Inner Membrane
HBNHX16	540	59.33	8.71	35.54	0.561	111.41	Inner Membrane

Table S3. The function of NHX gene in different plants and its effect on Na^+ and K^+ homeostasis under salt stress

Plant species	Gene name	Experimental method	Salt stress concentration	Changes of Na^+ content (compared with the control)	Changes of K^+ content (compared with the control)	K^+/Na^+
Nicotiana tabacum L.	*McNHX1*	Overexpression	200 mM NaCl	Leaf Na^+> 60 mg / g DW.	Leaf K^+ 40 mg / g DW (maintain normal)	relatively high
Oryza sativa L.	*OsNHX1*	Overexpression	150 mM NaCl	Na^+ decreases in the aboveground part	Aboveground K^+ rises	Significantly increased
Nicotiana tabacum L.	*TaNHX3*	Overexpression	150 mM NaCl	There was no significant difference in Na^+ in leaves.	Leaf K^+ rises.	Significantly increased
Ricinus communis L..	*SbNHX1*	Overexpression	200 mM NaCl	Na^+ content increased less.	The significant accumulation of K^+	Significantly increased
